# Fe^2+^ Alleviated the Toxicity of ZnO Nanoparticles to *Pseudomonas tolaasii* Y-11 by Changing Nanoparticles Behavior in Solution

**DOI:** 10.3390/microorganisms9112189

**Published:** 2021-10-20

**Authors:** Yuran Yang, Can Zhang, Kaili Li, Zhenlun Li

**Affiliations:** 1 Chongqing Key Laboratory of Soil Multiscale Interfacial Process, College of Resources and Environment, Southwest University, Chongqing 400716, China; yyran0322@email.swu.edu.cn (Y.Y.); zc1369@email.swu.edu.cn (C.Z.); 2 School of Chemical Engineering, University of Queensland, Brisbane, QLD 4072, Australia; kaili.li@uqconnect.edu.au

**Keywords:** *Pseudomonas tolaasii*, Ferrous iron, ZnO-NPs, detoxification, aerobic denitrification, magnesium

## Abstract

The negative effect of ZnO nanoparticles (ZnO-NPs) on the biological removal of nitrate (NO_3_^−^) has received extensive attention, but the underlying mechanism is controversial. Additionally, there is no research on Fe^2+^ used to alleviate the cytotoxicity of NPs. In this paper, the effects of different doses of ZnO-NPs on the growth and NO_3_^−^ removal of *Pseudomonas tolaasii* Y-11 were studied with or without Fe^2+^. The results showed that ZnO-NPs had a dose-dependent inhibition on the growth and NO_3_^−^ removal of *Pseudomonas tolaasii* Y-11 and achieved cytotoxic effects through both the NPs themselves and the released Zn^2+^. The addition of Fe^2+^ changed the behavior of ZnO-NPs in an aqueous solution (inhibiting the release of toxic Zn^2+^ and promoting the aggregation of ZnO-NPs), thereby alleviating the poisonous effect of ZnO-NPs on the growth and nitrogen removal of *P. tolaasii* Y-11. This study provides a theoretical method for exploring the mitigation of the acute toxicity of ZnO-NPs to denitrifying microorganisms.

## 1. Introduction

Nitrate (NO_3_^−^) is an increasingly serious pollutant in agricultural, urban, and industrial wastewater [1,2] due to the excessive use of fertilizers, discharge of livestock wastewater, and the infiltration of landfill leachate [3]. Previous reports demonstrated that nitrate could be removed efficiently and at a low-cost by microorganisms through assimilatory and dissimilatory reactions [4,5]. However, microorganisms are extremely susceptible to environmental factors. In recent decades, engineered nanoparticles (NPs), such as ZnO-NPs, have been widely used in sunscreens, coatings, and paints [6,7]. Commodities containing ZnO-NPs will inevitably be released into the environment during production, use and abandonment [6,8,9]. Finally, it accumulates and deposits in activated sludge [10]. Studies have shown that ZnO-NPs can change the community structure of functional flora in activated sludge [8,9,11] and inhibit enzyme activity [6], thus inhibiting nitrogen removal [12].

Up to now, many scholars found that Zn^2+^ dissolved from ZnO-NPs exhibited ion toxicity [6,10,13,14]. Zhang et al. [15] found that the accumulation of toxic Zn^2+^ in organisms resulted in the loss of 90% nitrogen removal capacity due to ZnO-NPs shock. In addition, ZnO-NPs are easily adsorbed on the surface of bacteria and can permeate into cells causing toxic effects [16]. Therefore, it is of great significance to clarify the toxicity source of ZnO-NPs in order to study and alleviate their toxicity.

Improving the stability of the ZnO-NPs or inhibiting their dissolution is one of the ways to alleviate the toxicity of the NPs. Some studies indicated that the composition of the solution had a key inhibitory effect on the dissolution of ZnO-NPs [6,17,18]. In addition, promoting aggregation and inhibiting diffusion can reduce the deposition of NPs on the surface of bacteria [19]. Iron is a widely available, environmentally friendly element, and Fe^2+^ is also an essential element for microbial growth. Some scholars proved that iron doping could reduce the cytotoxicity of NPs. For example, Li et al. [20] and Xia et al. [21] found that the iron matrix doped changed ZnO-NPs particles, slowing the rate of dissolution of the particles. Iron doping enhanced the combination of iron with zinc and oxygen, slowed the release of Zn^2+^, and achieved the purpose of reducing the cytotoxicity of ZnO [22]. However, there is no research on directly adding Fe^2+^ in sewage treatment to alleviate the cytotoxicity of ZnO-NPs.

Previous studies showed that Fe^2+^ could alleviate the cytotoxicity of CuO-NPs [23]. To determine the universality of Fe^2+^ to alleviate the cytotoxicity of NPs, we further explored the effect of Fe^2+^ on the cytotoxicity of ZnO-NPs. Using the scanning electron microscope (SEM), dynamic light scattering (DLS) and fourier transform infrared (FTIR) to further analyze the influence of exogenous Fe^2+^ on the water environment behavior of ZnO-NPs.

## 2. Materials and Methods

### 2.1. Bacterium and Culture Media

The used *P. tolaasii* Y-11 (KP410741) was isolated from winter paddy field, which proved that this strain was capable of NO_3_^−^ removal [24].

The basal medium (BM) consisted of the following components (1 L, pH 7.3): 0.31 g NaNO_3_, 2.56 g CH_3_COONa, 0.42 g Na_2_HPO_4_, 1.5 g KH_2_PO_4_ and 0.1 g MgSO_4_·7H_2_O. Fe^2+^ was added together with the substrate in the form of FeSO_4_·7H_2_O (0.05 g/L) to explore the alleviating effect of Fe^2+^ on the cytotoxicity of ZnO-NPs. The Luria-Bertani (LB) medium contained (1 L, pH 7.3) NaCl 10 g, tryptone 10 g, and yeast extract 5 g.

Each 250 mL conical flask contained 100 mL medium. The sterilizing conditions of the medium were as follows: 121 °C for 20 min.

### 2.2. ZnO-NP Preparation and Characterization

Purity ZnO-NPs (20 nm, 99.9%, Zewu Company, Chongqing, China) were used in this study. ZnO-NPs (100 mg) were dispersed in 50 mL ultrapure water, and the suspension was prepared by ultrasonic (600 W and 40 kHz) for 20 min according to the method previously studied [23]. The Zeta potential was measured with a Zeta potential analyzer (ZetaPlus, Brookhaven, NY, USA) (Table S1). The hydrodynamic diameter of ZnO-NPs at different concentrations were determined by Dynamic Light Scattering (DLS, Brookhaven, NY, USA) (Figure S1). 

### 2.3. Evaluation of Mg^2+^ and Fe^2+^ on NO_3_^−^ Removal Performance of Strain Y-11

Strain Y-11 was inoculated in LB medium, and shaken at 150 rpm (15 °C) for 36 h. Bacteria (10 mL) were harvested by centrifugation (6000 rpm, 5 min), washed twice with 5ml sterile ultrapure water, inoculated into different concentrations of Fe^2+^ basal medium (with or without Mg^2+^) and then incubated at 15 °C and 150 rpm [25]. All treatments were conducted in triplicates. The non-inoculation treatment was used as the control (CK); cell density (OD_600_) and nitrate (NO_3_^−^). Three parallel measurements were taken for each treatment.

### 2.4. Evaluation of Fe^2+^ on NO_3_^−^ Removal Performance of Strain Y-11 under ZnO-NPs (Zn^2+^) Stress

Strain Y-11 was exposed to BM with ZnO-NPs (0, 0.5, 5, 10 and 20 mg/L) or Zn^2+^ (0, 0.3, 0.6, 0.9 and 1.2 mg/L), and the non-inoculation treatment was used as the control (CK). The OD_600_, NO_3_^−^ and metal ions (Mg^2+^, Fe^2+^ and Zn^2+^) were investigated after 48 h of incubation. Three parallel measurements were taken for each treatment. 

### 2.5. Observation of ZnO-NPs Adsorption on Strain Y-11 Surface with or without Fe^2+^

The distribution of ZnO-NPs in bacterial suspension was analyzed by a scanning electron microscope (SEM, Phenom World, Eindhoven, Holland) and energy spectrum analysis (EDS). The treatment (5 mg/L ZnO-NPs) with significant difference in bacterial growth was selected, and 50 mL culture solution was centrifuged (6000 rpm, 10 min). The pellets were collected and fixed with 2.5% glutaraldehyde in 0.1 M phosphate-buffered solution (PBS, pH 7.4) at 4 °C for overnight. The treated supplies were washed again with PBS and dehydrated with a gradient of ethanol (50%, 70%, 80%, 90%, 15 min each). The sample was resuspended in 99% ethanol, dripped onto a silicon wafer, and dried in a dryer. Finally, the images were obtained using SEM-EDS at 15 kV voltage.

### 2.6. FTIR Analysis

In this study, FTIR was used to analyze the effect of Fe^2+^ addition on the main functional groups of the interaction between ZnO-NPs and strain Y-11. After 48 h of culture, the suspension treated with 5 mg/L ZnO-NPs was freeze-dried for 48 h. The 1 mg freeze-dried sample was ground with 100 mg potassium bromide in an agate mortar, and then pressed. FTIR (PerkinElmer, Waltham, MA, USA) spectrum was used to identify the main functional groups in the sample in the range of 400–4000 cm^−1^.

### 2.7. Analysis and Calculation

After 48 h of exposure, OD_600_ was tested using a spectrophotometer (UV755B, Shanghai Analytical Instruments General Factory, Shanghai, China) at an absorption wavelength of 600 nm. To eliminate the influence of high-concentration ZnO-NPs on the OD_600_ measurement, the following calculations were performed:

OD_600_ = OD_600T_ − OD_600CK_

where OD_600_ was the actual cell density, OD_600T_ and OD_600CK_ were the cell densities of the experimental group and the control group, respectively.

The medium (10 mL) was centrifuged at 8000 rpm for 5 min. The supernatant was used for determination of NO_3_^−^ and metal ions. Specifically, the measurement of the metal ions (Mg^2+^, Fe^2+^ and Zn^2+^) concentration used ICP-OES (5110, Agilent, Santa Clara, CA, USA). NO_3_^−^ was determined by hydrochloric acid spectrophotometry, with reference to He et al. [25].

The removal efficiency of NO_3_^−^ was calculated as follows:

R = (T_0_ − T_1_)/T_0_ × 100%

where R was the NO_3_^−^ removal efficiency (%), T_0_ and T_1_ represented the initial and final NO_3_^−^ concentration in the culture medium, respectively. The results were expressed as mean ± SD (standard deviation). All statistical analyses were carried out by one-way ANOVA.

## 3. Results

### 3.1. Effects of Fe^2+^ on Cell Proliferation and NO_3_^−^ Removal of P. tolaasii Y-11

To evaluate the optimal addition of Fe^2+^, we explored the influence of Fe^2+^ on the NO_3_^−^ removal of strain Y-11 (Figure S2). The cell proliferation and NO_3_^−^ removal performance almost ceased when Mg^2+^ was not added to the BM. Even with the addition of Fe^2+^, the improvement was only slight. Therefore, Mg^2+^ was an essential nutrient of the growth and metabolism of strain Y-11 [25,26]. When the Fe^2+^ concentration increased from 0 to 10 mg/L, the OD_600_ significantly increased from 1.41 to 1.95, and the NO_3_^−^ removal efficiency significantly increased from 58.66% to 85.89%. After that, the growth and NO_3_^−^ removal showed no significant changes regarding the increase in the amount of Fe^2+^. So, the amount of Fe^2+^ added was 10 mg/L.

### 3.2. Effects of Fe^2+^ on Cell Proliferation and NO_3_^−^ Removal of P. tolaasii Y-11 under ZnO-NPs Stress

**Figure 1:**
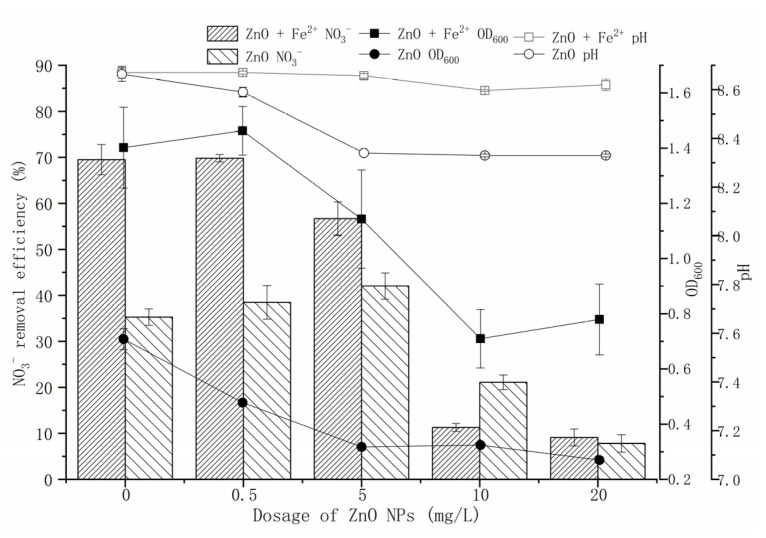
Effect of Fe^2+^ on the proliferation and NO_3_^−^ removal of *P. tolaasii* Y-11 under ZnO-NPs stress.

In the Fe^2+^-free treatment, ZnO-NPs had an inhibitory effect on the growth of strain Y-11, showing a dose-dependent pattern (Figure 1). ZnO-NPs, in measurements of 0.5 mg/L and 5 mg/L, promoted the NO_3_^−^ removal (35.48% and 47.63%, respectively); 10 mg/L and 20 mg/L ZnO-NPs inhibited the NO_3_^−^ removal (21.10% and 7.81%, respectively) (0 mg/L ZnO-NPs treatment was 35.26%). Although low-content ZnO-NPs inhibited cell proliferation, it promoted denitrification-related enzyme activity. It may be that the adsorption of ZnO-NPs on the surface of strain Y-11 inhibited cell proliferation due to electrostatic attraction [16]. However, the Zn^2+^ released from the low-content ZnO-NPs did not exceed the threshold of toxicity of strain Y-11, but stimulated the related enzyme activity. As the content of ZnO-NPs further increased, the cell activity was seriously affected. In the Fe^2+^-containing treatment, as the ZnO-NPs content increased from 0.5 mg/L to 20 mg/L, the NO_3_^−^ removal efficiencies decreased from 69.82% to 9.10%, and the OD_600_ decreased from 1.463 to 0.779. At the same ZnO-NPs concentration, the NO_3_^−^ removal efficiencies and OD_600_ of Fe^2+^-containing treatment were conspicuously higher than those of the Fe^2+^-free treatment (0-5 mg/L ZnO-NPs), which implied that Fe^2+^ could alleviate the cytotoxicity of ZnO-NPs. The reason for this could be that Fe^2+^ was a coenzyme factor of microbial metalloproteinases or certain functional enzymes, which could promote the electron transfer of microorganisms, improve the activity of microorganisms, and improve the utilization and transformation of nitrogen [27,28,29]. Additionally, with a high content of ZnO-NPs, especially 20 mg/L ZnO-NPs, the addition of Fe^2+^ did not significantly promote the removal of the NO_3_^−^ by strain Y-11. We believed that 10 mg/L Fe^2+^ was not enough to alleviate all the cytotoxicity of ZnO-NPs under the treatment of this content of ZnO-NPs. Therefore, the cytotoxicity of ZnO-NPs could be further alleviated by increasing the dosage of Fe^2+^ (after all, within the range of 100 mg/L Fe^2+^, there was no ion toxicity to strain Y-11 (Figure S2)).

### 3.3. Effect of Fe^2+^ on the Release of Zn^2+^ from ZnO-NPs

**Figure 2:**
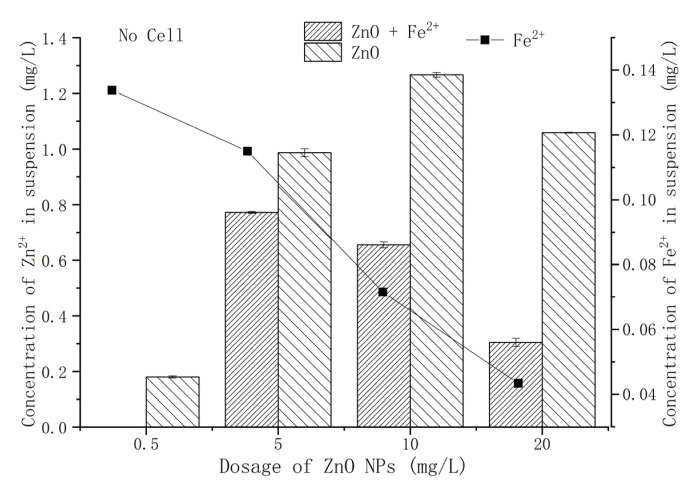
Effect of different content of ZnO-NPs on Zn^2+^ release.

It has been substantially recognized that ZnO-NPs releases Zn^2+^ in aqueous environments [14,30]. Through static dissolution experiments, the effect of Fe^2+^ on the amount of Zn^2+^ release from ZnO-NPs was evaluated (Figure 2). With the increase in ZnO-NPs content, the dissolved Zn^2+^ increased first and then decreased, reaching the maximum release of Zn^2+^ (1.27 mg/L) at 10 mg/L ZnO-NPs. This was similar to the results of Wang et al. [31] and Huang et al. [26]. The rapid aggregation and precipitation of high-content ZnO-NPs led to a reduction in Zn^2+^ dissolution [32]. The exogenous Fe^2+^ suppressed the release of Zn^2+^, and the release of Zn^2+^ reached the maximum value (0.77 mg/L) when treated with 5 mg/L ZnO-NPs.

### 3.4. Effect of Fe^2+^ on Cell Proliferation and NO_3_^−^ Removal of P. tolaasii Y-11 under Zn^2+^ Stress

**Figure 3:**
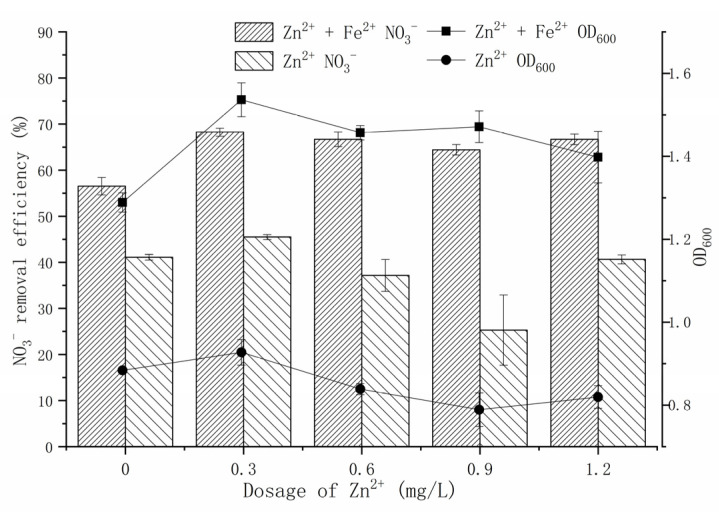
Effect of Fe^2+^ on the proliferation and NO_3_^−^ removal of *P. tolaasii* Y-11 under Zn^2+^ stress.

Previous studies have shown that the release of Zn^2+^ from ZnO-NPs is the main source of its toxicity to certain microorganisms in aqueous media [6,33]. To investigate the source of the cytotoxicity of ZnO-NPs, a Zn^2+^ simulation experiment was conducted according to the amount of Zn^2+^ released from ZnO-NPs (Figure 3). Zn^2+^ is an important micronutrient, and an appropriate amount of Zn^2+^ can promote the growth and metabolism of microorganisms. An amount of 0.3 mg/L Zn^2+^ had a positive effect on strain Y-11. In the Fe^2+^-free treatment, the amount of Zn^2+^ released by 0.5–5 mg/L ZnO-NPs was in the range of 0.18–0.99 mg/L (Figure 2). In this case, the growth of strain y-11 was inhibited, but NO_3_^−^ removal was promoted (Figure 1). Intriguingly, in the Fe^2+^-free treatment, as the Zn^2+^ concentration increased (0.3–0.9 mg/L), the OD_600_ decreased, and the NO_3_^−^ removal efficiency decreased from 45.49% to 25.28%. The release of Zn^2+^ from ZnO-NPs is a slow process, which causes the difference between the results of the Zn^2+^ simulation experiments and the ZnO-NPs experiment. However, it was worth affirming that the Zn^2+^ released from low content ZnO-NPs (0.5 mg/L and 5 mg/L) was the main source of its cytotoxicity. The amount of Zn^2+^ released from 10 mg/L ZnO-NPs was 1.27 mg/L, and the strain growth and NO_3_^−^ removal efficiency were inhibited (Figure 2 and Figure 3). Furthermore, when the content of Zn^2+^ was 1.2 mg/L, the NO_3_^−^ removal efficiency increased to 40.65%. The reasons for this need further study. When the content of ZnO-NPs was 20 mg/L, although the release of Zn^2+^ was reduced, the activity of the strain was significantly inhibited (Figure 1 and Figure 2). The results showed that the high content of ZnO-NPs was the main source of their cytotoxicity.

In the Fe^2+^-containing treatment, exogenous Fe^2+^ could effectively alleviate the toxicity of Zn^2+^ and maintain a high OD_600_ (1.4) and NO_3_^−^ removal efficiency (65%). Prominently, strain Y-11 was more affected by the content of ZnO-NPs than the content of Zn^2+^ (Figure 1). This further proved that ZnO-NPs themselves and Zn^2+^ interact together to cause their cytotoxicity to strain Y-11. This result matched the findings in another study which reported that the content of ZnO-NPs exceeded 1 mg/L, and the source of its toxicity to bacteria was not only due to dissolved Zn^2+^, but also contained ZnO-NPs themselves [26]. However, Wang et al. [31] believed that the cytotoxicity of ZnO-NPs was only dependent on Zn^2+^. The sources of cytotoxicity of ZnO-NPs varied with different strain solutions, but dissolved Zn^2+^ was part of this source of cytotoxicity. The exogenous Fe^2+^ could alleviate the cytotoxicity of ZnO-NPs.

### 3.5. Effects of Fe^2+^ on the Main Functional Groups of the Interaction between ZnO-NPs and Strain Y-11

**Figure 4:**
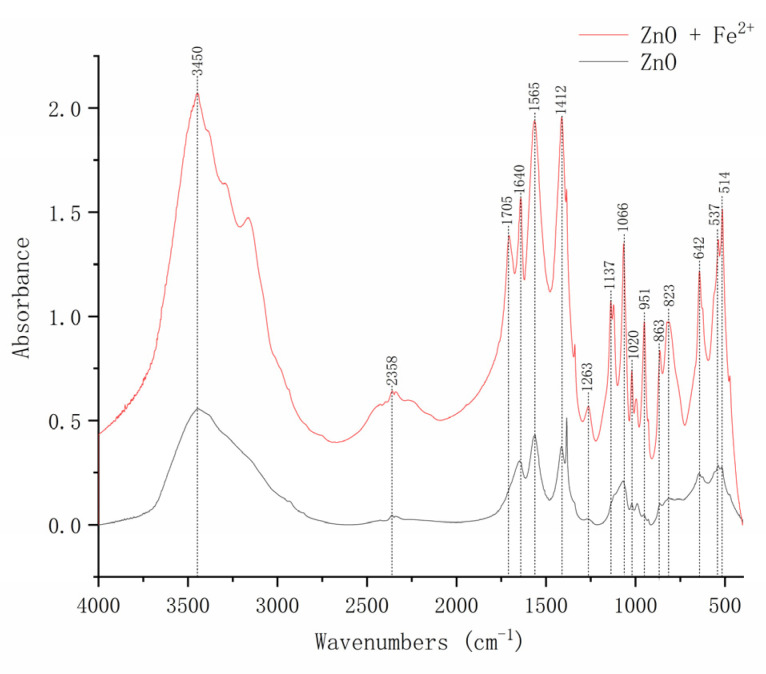
FTIR spectra of 5 mg/L ZnO-NPs with or without Fe^2+^ addition.

In order to further clarify the role of Fe^2+^ in alleviating the cytotoxicity of ZnO-NPs, the effects of Fe^2+^ on the surface functional groups of ZnO-NPs were studied by FTIR (Figure 4). The peak value appeared at 537 cm^−1^, confirming the presence of ZnO. Previous studies showed that the peaks in the range of 400–800 cm^−1^ were related to the metal-oxide group. Therefore, we believed that the newly appearing peak at 514 cm^−1^ was an Fe-O single peak in the Fe^2+^-containing treatment. A sharp peak appeared at 642 cm^−1^, corresponding to the stretching vibration of ZnO [34]. The absorption peaks at 823 cm^−1^ were the stretching vibrations of the unsaturated carbon–hydrogen bonds on the benzene ring. The peak observed at 863 cm^−1^ was considered to be the Zn-OH bending vibration [35]. The peaks at 1020 and 1066 cm^−1^ belonged to the P-O symmetric stretching vibration [20]. This indicated that the phosphate in the medium reacted with Zn or adsorbed on the surface of ZnO-NPs. The peaks at 2358 cm^−1^ were due to the sample’s absorption of CO_2_ from the environment. The absorption peaks at 3450 cm^−1^ were due to O-H stretching [36]. The fluctuation of the wavenumber of ZnO-NPs after the addition of Fe^2+^ showed that Fe^2+^ played a role in alleviating the cytotoxicity of ZnO-NPs. This confirmed that the addition of Fe^2+^ played a role in alleviating the cytotoxicity of ZnO-NPs.

### 3.6. Effects of Fe^2+^ on the Hydrodynamic Diameter of ZnO-NPs

The hydrodynamic diameter reflected the aggregation state of ZnO-NPs in the solution. The hydrodynamic diameter of ZnO-NPs increased from 687.21 nm to 1540.4 nm as its content increased (Table 1). That is, the high content of ZnO-NPs greatly increased the chance of a collision between the NP, resulting in the agglomeration of ZnO-NPs. In this study, exogenous Fe^2+^ made the hydrodynamic diameter of ZnO-NPs reach the micron level, losing its original specificity. It could be seen that the addition of Fe^2+^ promoted the aggregation of ZnO-NPs and slowed the diffusion of ZnO-NPs, thus slowing down the cytotoxicity of ZnO-NPs.

### 3.7. Effects of Fe^2+^ on the Adsorption of ZnO-NPs on the Surface of Strain Y-11

**Figure 5:**
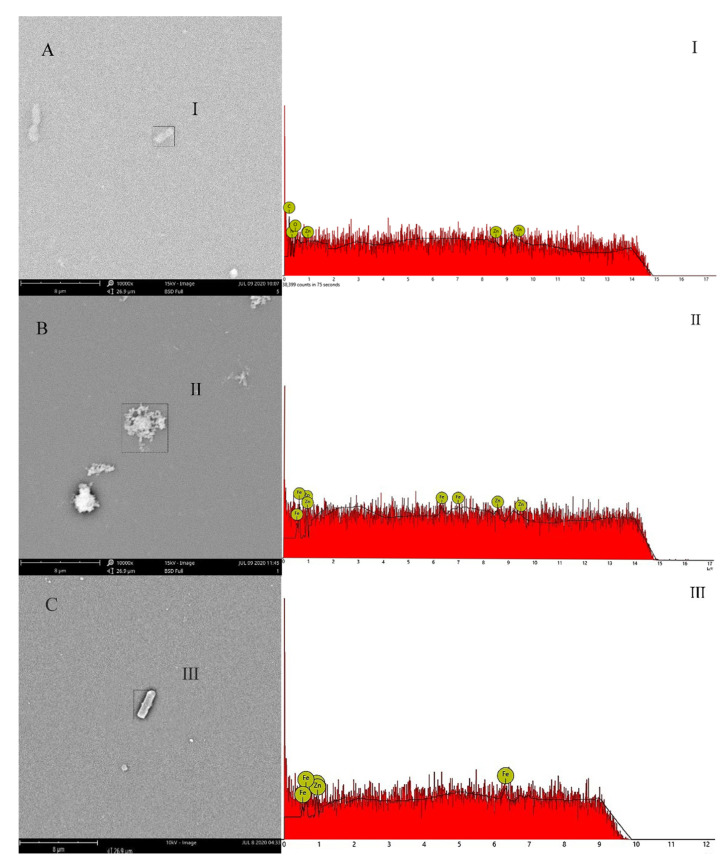
SEM-EDS images of Y-11 exposed to 5 mg/L ZnO-NPs with or without Fe^2+^. (**A**) is Fe^2+^-free treatment; (**B**) is Fe^2+^ adsorbed on ZnO-NPs; (**C**) is Fe^2+^-containing treatment.

The evidence demonstrated that the large specific surface area made NPs easy to attach to microbial flocs through processes such as adsorption [16]. SEM-EDS images showed that the ZnO-NPs were adsorbed on the surface of the bacteria when Fe^2+^ was not added to the medium (Figure 5). The EDS spectrum showed that there was a strong Zn peak intensity near 1, 8.6, 9.5 keV, and an oxygen peak at 0.6 keV, indicating that ZnO-NPs were adsorbed on strain Y-11. Studies showed that ZnO-NPs were first deposited on the cell surface under the action of electrostatic force, resulting in a decrease in cell growth [6]. This study also found that ZnO-NPs exposure treatment could lead to a reduction in bacterial growth (Figure 1). The exogenous Fe^2+^ promoted the adsorption of Fe^2+^ on the surface of ZnO-NPs, leading to the formation of a “coating” on the surface of ZnO-NPs (Figure 5B). Additionally, the decrease in Zeta potential indicated that the addition of Fe^2+^ reduced the electrostatic repulsion between the ZnO-NPs (Table S1) and reduced the contact between the ZnO-NPs and the bacteria (Figure 5C). In summary, exogenous Fe^2+^ reduced the toxicity of NPs due to the increase in electrostatic repulsion and the “coating effect” of Fe^2+^ on the surface of ZnO-NPs, which hindered the physical contact between ZnO-NPs and the cell membranes. 

## 4. Discussion

In this study, by comparing the effects of ZnO-NPs and Zn^2+^ on the strain Y-11, it was concluded that the toxicity of ZnO-NPs to strain Y-11 came from the NPs themselves and the dissolved Zn^2+^. This was consistent with the research results of Huang et al. [26]. On the one hand, ZnO-NPs itself could contact the cell and destroy the cell membrane. Due to its small size, ZnO-NPs had a good dispersibility and strong penetrating ability in an aqueous solution [37], could enter cells and cause a toxic effect on strain Y-11. On the other hand, the Zn^2+^ dissolved from ZnO-NPs could produce heavy metal ion toxicity to the strain Y-11. Related studies found that when the Zn^2+^ content exceeded 0.5 mg/L, it had a significant inhibitory effect on the biological nitrogen removal [25]. The nano-toxicity and Zn^2+^ toxicity of ZnO-NPs were affected by its dissolution, aggregation, adsorption and other behavior in the water. In this sense, the cytotoxicity of NPs could be mitigated by changing their behavior in solution. 

Firstly, the release of free Zn^2+^ was an aspect of ZnO-NPs cytotoxicity. Studies showed that Zn^2+^ released from ZnO-NPs could severely inhibit denitrification [13,14]. For example, Zhang et al. [15] found that when toxic Zn^2+^ accumulated in organisms, 90% of the denitrification capacity was lost due to ZnO-NPs shock. Similarly, related studies found that Zn^2+^ released from ZnO-NPs had a toxicity effect on purely cultured microorganisms (*Escherichia coli*, *Vibrio fischeri*, etc.) [38]. ZnO-NPs had a good adsorption property, and the dissolved Zn^2+^ was adsorbed on its surface. This caused the dissolved Zn^2+^ to infiltrate into the cells along with the ZnO-NPs, which could be toxic to cells [16]. Li et al. [20] reduced the cytotoxicity of ZnO-NPs by adding Zn^2+^ chelating agents (citrate ion, EDTA, etc.). Wirth et al. [39] found that humic acid(HA) could lead to the reduction in free Ag^+^ and reduce the toxicity of Ag-NPs. This study found that the direct addition of Fe^2+^ could effectively reduce the dissolution of Zn^2+^ (Figure 2). It was noticed that the content of Fe^2+^ in the solution decreased from 0.44 mg/L to 0.04 mg/L with the increasing ZnO-NPs dose. It showed that Fe^2+^ was adsorbed by ZnO-NPs and inhibited the dissolution of Zn^2+^. 

In addition, ZnO-NPs itself could also cause cytotoxicity. The small size of NPs predicted a greater mobility and cell penetration [40], and their aggregation state in an aqueous solution affected their mobility and toxicity [19,41]. Hence, reducing the adsorption of NPs on the cell surface and uptake could alleviate their cytotoxicity. Exogenous Fe^2+^ compressed the electric double layer on the surface of ZnO-NPs [42], thus promoting the aggregation of NPs and effectively reducing the mobility of ZnO-NPs. The aggregated ZnO-NPs reduced the deposition on the surface of bacteria and reduced cell uptake. Similarly, adding fulvic acid (FA) could prevent the physical contact between CuO-NPs and the cell membrane [43]. However, this approach would increase the burden of sewage treatment. In this study, the direct addition of Fe^2+^ to alleviate the cytotoxicity of ZnO-NPs provided theoretical support for the emergency regulation of the impact of NPs on the biological nitrogen removal system. 

## Figures and Tables

**Table 1:** Effect of Fe^2+^ on the hydrodynamic diameter of ZnO-NPs.

Particle Size (nm)		ZnO-NPs Content (mg/L)		
	0.5	5	10	20
Fe^2+^-free	--	687.21	973.76	1540.4
Fe^2+^-containing	3103.72	4039.37	4583.63	4396.22

-- means not detected.

## Data Availability

Not applicable.

## Supplementary Materials

The following are available online at https://www.mdpi.com/article/10.3390/microorganisms9112189/s1, Figure S1: Hydrodynamic diameter of ZnO-NPs (1000 mg/L), Figure S2: Effects of Fe^2+^ on the growth and NO_3_^−^ removal of *P. tolaasii* Y-11, Table S1: Zeta potential of ZnO-NPs with and without Fe^2+^.
