# Supplementary material for: Fe2+ Alleviated the Toxicity of ZnO Nanoparticles to Pseudomonas tolaasii Y-11 by Changing Nanoparticles Behavior in Solution
Source: Microorganisms. 2021 Oct 20;9(11):2189. doi: 10.3390/microorganisms9112189 (PMC8620691; doi:10.3390/microorganisms9112189)
Supplement: Supplementary file 1 [file microorganisms-09-02189-s001.zip › microorganisms-1341891-supplementary.pdf]

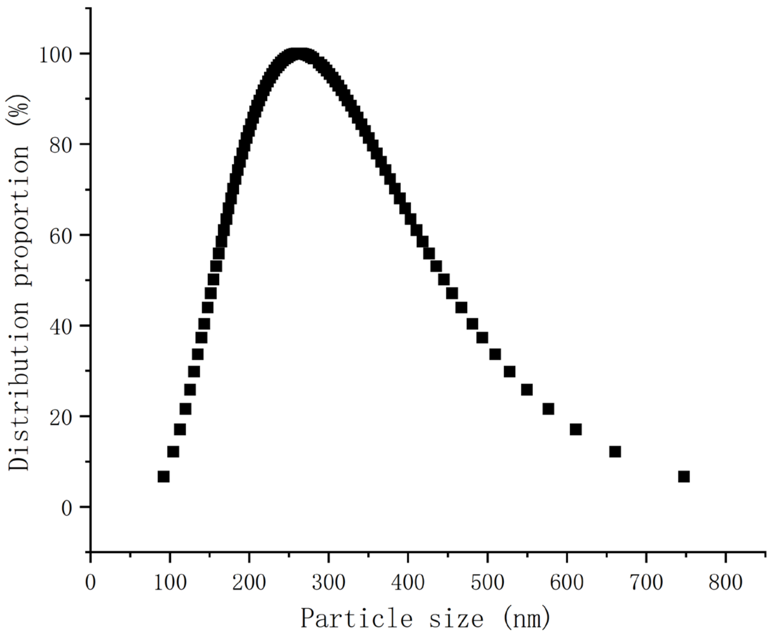

Figure S1. Hydrodynamic diameter of ZnO-NPs (1000 mg/L)

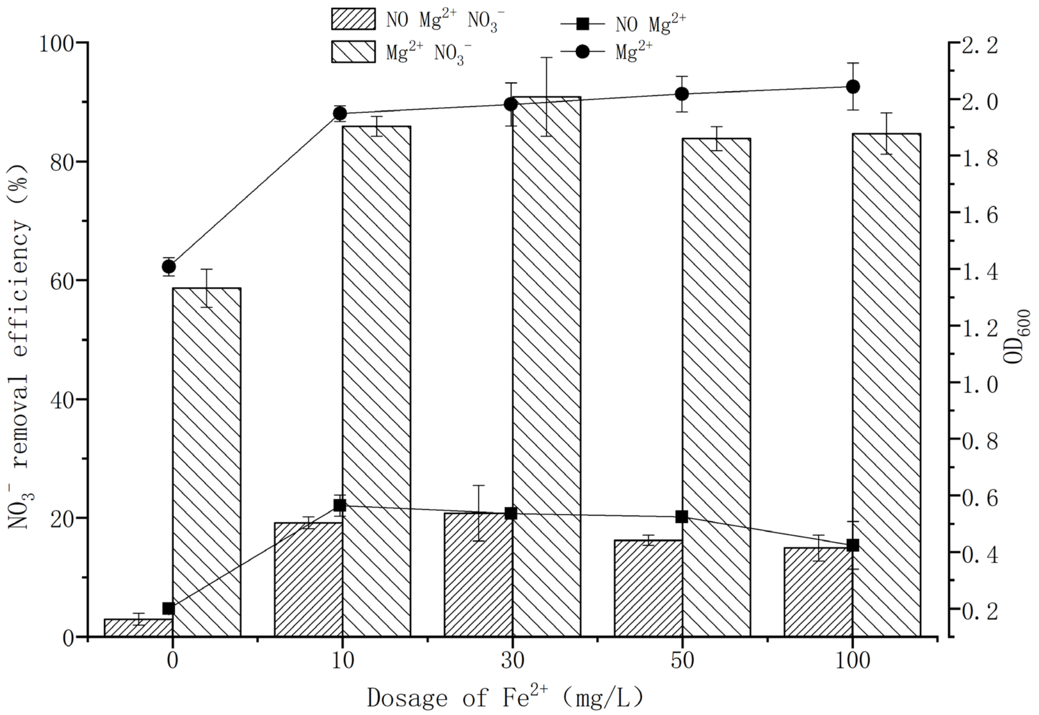

Figure S2. Effect of Fe^{2+} on the growth and NO_3^- removal of *P. tolaasii* Y-11.

Table S1. Zeta potential of ZnO-NPs with and without Fe^{2+}

	**Zeta Potential (mV)**
Strain Y-11	−16.88 ± 1.75
200 mg/L ZnO-NPs	11.15 ± 1.9
200 mg/L ZnO-NPs + Fe^{2+}	6.27 ± 1.86

The data are expressed as mean ± SE (n = 8)

1
